# Comparison of robot-assisted retroperitoneal laparoscopic adrenalectomy versus retroperitoneal laparoscopic adrenalectomy for large pheochromocytoma: a single-centre retrospective study

**DOI:** 10.1186/s12893-020-00895-5

**Published:** 2020-10-07

**Authors:** Sheng-Qiang Fu, Chang-Shui Zhuang, Xiao-Rong Yang, Wen-Jie Xie, Bin-Bin Gong, Yi-Fu Liu, Ji Liu, Ting Sun, Ming Ma

**Affiliations:** 1 Department of Urology, the First Affiliated Hospital of Nanchang University, Nanchang, 330000 Jiangxi Province China; 2 Union Shenzhen Hospital, Huazhong University of Science and Technology, Shenzhen, 518052 China

**Keywords:** Pheochromocytoma, Robotic surgery, Laparoscopic adrenalectomy

## Abstract

**Background:**

To evaluate the feasibility and safety of robot-assisted retroperitoneal laparoscopic adrenalectomy (RARLA) for large pheochromocytomas (PHEOs; size≥6 cm) compared with retroperitoneal laparoscopic adrenalectomy (RLA).

**Methods:**

Fifty-one patients who underwent adrenalectomy for large PHEOs between March 2016 and January 2019 were enrolled and divided into two groups, including 32 RLA cases and 19 RARLA cases. We compared the perioperative efficacy and long-term follow-up results between the two groups.

**Results:**

Preoperative data, including demographics, comorbidities and tumour characteristics, were similar between the groups. Intraoperatively, the RARLA group had a lower incidence of haemodynamic instability (26.3% vs. 56.2%, *P* = 0.038) and less intraoperative blood loss (100 ml vs. Two hundred milliliter, *P* = 0.042) than the RLA group. The groups showed no significant differences in operative time or transfusion rates. Postoperatively, the time to diet resumption, time to ambulation, time to drainage removal and postoperative hospital stay were shorter in the RARLA group than in the RLA group (1 d vs. 2 d, *P* = 0.027; 1 d vs. 2 d, *P* = 0.034; 3 d vs. 5 d, *P* = 0.002; 5 d vs. 6 d, *P* = 0.02, respectively). The groups exhibited no significant differences in the duration of anaesthetic use, complications, or long-term follow-up results for the blood pressure (BP) improvement rate.

**Conclusions:**

Compared with RLA, RARLA is a safe, feasible and even optimized procedure for large PHEOs.

## Background

Pheochromocytoma (PHEO) is a neuroendocrine tumour mainly originating from the adrenal medulla, and surgical excision of the lesion is the main treatment [1]. Surgical resection of PHEO is a challenging procedure. In addition to the risks associated with general adrenal surgery, perioperative haemodynamic fluctuations have always been a nonnegligible risk during surgery for PHEO because they can lead to serious cardiovascular and cerebrovascular complications and even perioperative mortalities [2].

The management of large PHEOs is more challenging than that of small PHEOs because large tumours have been shown to be significantly associated with potential malignancy, haemodynamic instability and severe morbidity. In the past, open surgery has been the first choice for resection of PHEO. It was not until 1992 that laparoscopy was gradually applied in the treatment of PHEO [3]. Several subsequent studies have confirmed the safety and feasibility of laparoscopic surgery for PHEO, [4–6] which has led to the widespread use of laparoscopic surgery for PHEO resection.

At present, laparoscopic adrenalectomy mainly includes retroperitoneal and transabdominal approaches. Several studies have suggested that retroperitoneal laparoscopic adrenalectomy (RLA) is superior to or at least comparable to transabdominal laparoscopic adrenalectomy for PHEO, even a large PHEO (size ≥6 cm), in terms of operation feasibility and postoperative recovery [5, 7–9]. Although robot-assisted laparoscopic adrenalectomy for PHEO has been reported in recent years, reports of robot-assisted laparoscopic adrenalectomy for large PHEO, a special type of PHEO, are lacking. The aim of this retrospective study was to compare the safety and feasibility between RARLA and RLA.

## Methods

### Patient baseline characteristics

All data for patients who underwent robot-assisted adrenal resection or retroperitoneal laparoscopic adrenal resection between March 2016 and January 2019 were extracted from the electronic medical record system maintained in the Urology Department of the First Affiliated Hospital of Nanchang University. The inclusion criteria were a unilateral, localized adrenal lesion and a radiographic largest axial tumour diameter ≥ 6 cm; clinical symptoms, imaging examinations and laboratory tests of adrenal hormones supported the diagnosis of pheochromocytoma in the patients included in this study. The American Association of Anesthesiologists (ASA) classification was I-IV, and the surgical risk score according to the National Nosocomial Infections Surveillance (NNIS) was 0–4 [1]. Patients with bilateral pheochromocytoma or pheochromocytoma located outside the adrenal area were excluded. The surgical method was decided by the surgeon after discussion with the patient and the patient’s family. A total of 51 procedures were performed, including 32 patients undergoing retroperitoneal laparoscopic pheochromocytoma resection (RLA, *n* = 32) and 19 patients undergoing robot-assisted retroperitoneal laparoscopic pheochromocytoma resection (RARLA, *n* = 19). All patients signed informed consent before surgery, and the study protocol was approved by the Institutional Review Board of the First Affiliated Hospital of Nanchang University.

### Preoperative preparation

All patients were treated with phenoxybenzamine or terazosin for at least 2 weeks before surgery, and patient with tachycardia (heart rate > 100 beats per minute) were treated with metoprolol, a receptor blocker. All patients were rehydrated 3 to 5 days before surgery through intravenous infusion of crystals/colloid. Surgery will not be scheduled until the patient meets the following criteria: stable BP at less than 140/90 mmHg, a heart rate less than 100 beats per minute, and haematocrit less than 0.45.

### Surgical procedure of RARLA

All the operations were performed by three surgical groups in the Urology Department of the First Affiliated Hospital of Nanchang University, three of whom had at least 5 years of robotic surgery experience and longer laparoscopic surgery experience, and all possessed the title of chief physician, reflecting their excellent technical skills. The anaesthesiologists were also experienced physicians with the title chief physician.

**Fig. 1:**
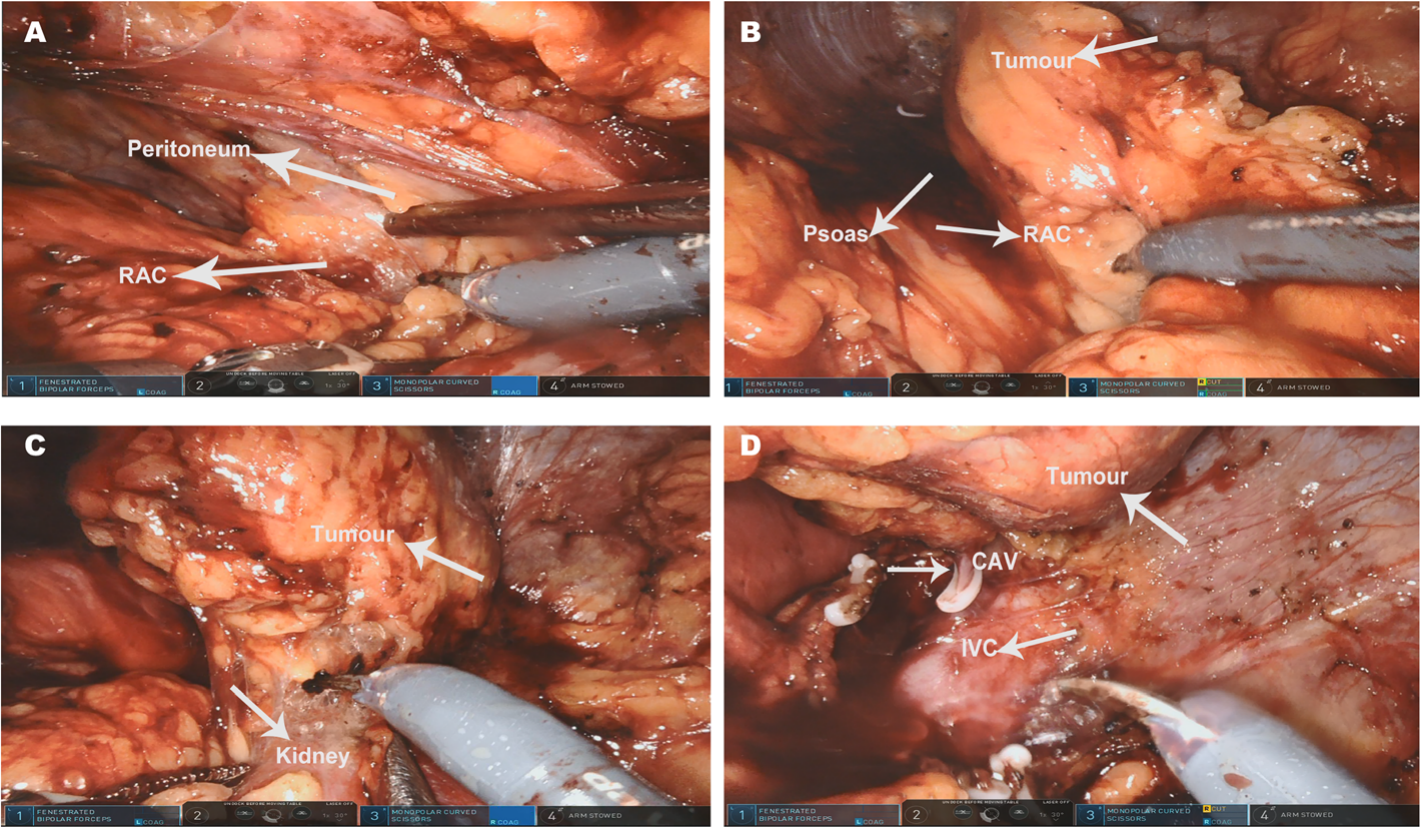
Representative intraoperative images of a patient with a 7-cm right-sided PHEO who underwent RARLA. **a** The first anatomical layer (between the peritoneal reflex and RAC), **b** the second anatomical layer (between the dorsal kidney and the psoas major), **c** the third anatomical layer (between the upper pole of the kidney and the adrenal gland), and **d** ligation and disconnection of the CAV are shown in the intraoperative photo. CAV, Central adrenal vein; IVC, Inferior vena cava; PHEO, Pheochromocytoma; RAC, Renal adipose capsule; RARLA, Robot-assisted retroperitoneal laparoscopic adrenalectomy

RARLA procedures in this study were performed with the Da Vinci SI Surgical System (Intuitive Surgical, Sunnyvale CA, USA). The retroperitoneal cavity was first established, and then the cannula was placed and the retroperitoneal fat was removed. The Gerota fascia was cut diagonally in the direction of the psoas major close to the psoas major, extending above the diaphragm and below the upper margin of the iliac fossa. Then, the Gerota fascia was opened on the outside of the peritoneal reflex to expose the renal adipose capsule. The avascular space between the upper pole of the kidney fat sac and the peritoneal reflex was separated upward to the subphrenic space with care to avoid damage to the peritoneum, which is the first anatomical plane (Fig. 1a). The separation procedure was performed between the dorsal pole of the kidney and the psoas major (the second anatomical plane), with separation upward to the lower diaphragm (Fig. 1b). The renal adipose capsule was opened against the upper pole of the kidney, and the layer between the upper pole of the kidney and the adrenal gland was separated to depth, which is the third anatomical level (Fig. 1c). After the perirenal fat was removed, a large adrenal tumour could be observed and connected to the central adrenal vein. The adrenal tumour was removed completely after the central vein was clipped with HEM-O-lock (Fig. 1d).

### Surgical procedure of RLA

RLA procedures were performed with full HD (High definition) laparoscopic operating instruments. The retroperitoneal cavity was first prepared, and then the cannula was placed. Next, the retroperitoneal fat was cleaned: all extraperitoneal fat was separated from top to bottom, and the fat was turned over and placed into the iliac fossa, revealing important anatomical markers such as the perirenal fascia and retroperitoneal reflex. The perirenal fascia was then longitudinally incised to the lower diaphragm and the upper margin of the iliac fossa. The adrenal tumour was then exposed and completely removed at three anatomical levels as described above in the RARLA procedure.

### Baseline data and outcome indicators

The preoperative data of the patients, including demographic characteristics (age, sex and body mass index), comorbidities (ASA, NNIS, hypertension, coronary heart disease [CHD], stroke, diabetes and arrhythmia), and tumour characteristics (tumour laterality and size), were collected. The indicators recorded during the operation were haemodynamic instability (HI), operative time, estimated blood loss (EBL) and blood transfusion rate. After surgery, the patients’ morbidity (overall morbidity, severe morbidity and CVD morbidity), recovery procedure (the duration of anaesthetic use, the time to oral food intake, the time to ambulation, the time to removal of the drainage tube and postoperative hospitalization days) and long-term follow-up results for the BP improvement rate were recorded.

HI was defined as BP greater than 180 mmHg or a mean arterial pressure less than 60 mmHg [10]. EBL was calculated as the amount of fluid in the aspirator minus the amount of fluid washed plus the amount of fluid soaked in gauze. Postoperatively, the classification of morbidity conforms to the Clavien-Dindo classification criteria [11]. Severe morbidity was defined as Clavien-Dindo III or IV. CVD morbidity mainly refers to complications associated with CVD, such as postoperative hypotension requiring the use of vasopressors or blood transfusion, myocardial infarction, stroke, atrial fibrillation or ventricular fibrillation, and thrombotic complications (pulmonary embolism or deep vein thrombosis). BP improvement was defined as a decrease in BP after surgery compared with that before surgery or a decrease in the dose or type of antihypertensive drugs after surgery.

### Statistical analysis

All data was analysed using SPSS 20.0 (SPSS, Inc., Chicago, IL, USA). Continuous variables with a normal distribution were expressed as the mean ± standard deviation, and the independent-sample Student’s t-test was used to analyse differences between the groups. If continuous variables did not conform to a normal distribution, they were presented with the median (interquartile range), and differences between groups were tested with the Mann-Whitney U test. Categorical variables were presented by a number (percentage), and Pearson’s chi-square test was used to detect differences between groups. *P* < 0.05 was considered statistically significant.

## Results

The patients’ baseline data are presented in Table 1. No statistically significant differences in demographic characteristics, comorbidities and tumour characteristics were found between the RARLA group and the RLA group. Table 2 shows the intraoperative and postoperative outcomes of the patients. Intraoperatively, the incidence of HI in the RARLA group was lower than that in the RLA group (26.3% vs 56.2%, *P* = 0.038), and EBL was lower in the RARLA group than in the RLA group (100 ml vs 200 ml, *P* = 0.042). No significant difference in operative time was noted between the two groups. Intraoperative transfusion rates were higher in the RLA group than in the RARLA group (21.9% vs 5.3%), with no statistical difference (*P* = 0.238). Postoperatively, the incidence of morbidity and the duration of anaesthetic use were similar in both groups. The time to oral food intake, the time to ambulation, the time to removal of the drainage tube and postoperative hospitalization days were shorter in the RARLA group than in the RLA group, and the difference was statistically significant (1 d vs 2 d, *P* = 0.027; 1 d vs 2 d, *P* = 0.034; 4 d vs 5 d, *P* = 0.002 and 5 d vs 6 d, *P* = 0.02, respectively). The BP improvement rate was similar between the two groups in the long-term follow-up (88.9% vs. 90.0%, *P* = 0.7144).

**Table 1:** Preoperative profiles of patients in this study

Variable	RLA (*n* = 32)	RARLA (*n* = 19)	*p* value
Demographic characteristics			
Age(years)	47.53 ± 14.048	44 ± 9.062	0.332&
Gender			
Female	17(53.1)	8(42.1)	0.447
Male	15(46.9)	11(57.9)	
BMI (kg/m2)	25.83 ± 4.45	26.64 ± 3.82	0.5116&
Comorbidities			
ASA score			
I/II	7(21.9)	1(5.3)	0.238
III/IV	25(78.1)	18(94.7)	
NNIS			
0/1	23(71.9)	12(63.2)	0.517
2/3	9(28.1)	7(36.8)	
Coronary heart disease (CHD)	8(25.0)	5(26.3)	0.917
Hypertension	30(93.75)	18(94.74)	0.6379
Diabetes	16(50.0)	9(47.4)	0.856
Arrhythmia	10(31.2)	7(36.8)	0.682
Tumor characteristics			
Laterality			
Left	16(50)	9(47.4)	0.856
Right	16(50)	10(52.6)	
Diameter of tumor	7.65(6.625, 9)	8(6, 9)	0.859§

Continuous normally distributed variables were expressed as the mean ± standard deviation (SD); Continuous non-normally distributed variables were expressed as the median (interquartile range); categorical variables are expressed as the number (percentage). Independent sample t-test was used to compare the differences between the means of two continuous normally distributed variables and Mann-Whitney U test was used to compare the differences between the means of two continuous non-normal distribution variables. Chi-square test was used to compare the differences between the categorical variables

*&* Independent sample t-test, *§* Mann-Whitney U test, *ASA* American Society of Anesthesiologists, *BMI* Body mass index, *NNIS* National Nosocomial Infections Surveillance *RLA* Retroperitoneal laparoscopic adrenalectomy, *RARLA* Robotic assisted retroperitoneal laparoscopic adrenalectomy

**Table 2:** Perioperative and prognosis data of patients in this study

Variable	RLA (*n* = 32)	RALA (*n* = 19)	*p* value
**Intraoperative parameters**			
HI	18(56.2)	5(26.3)	0.038
Operating time (min)	165.0 ± 69.5	166.3 ± 54.0	0.944&
EBL (mL)	200(80,300)	100(50,200)	0.042§
Transfusion [n (%)]	7(21.9)	1(5.3)	0.238
**Postoperative data**			
Overall morbidity [n (%)]	9(28.1)	6(31.6)	0.794
Severe morbidity [n (%)](Clavien III/IV)	2(6.25)	1(5.3)	0.6379
CVD morbidity	6(18.8)	2(10.5)	0.702
Duration of anesthetic use(days)	3.78 ± 1.601	2.95 ± 1.682	0.084 &
Time to oral food(days)	2(1.25, 3)	1(1, 2)	0.027§
Time to ambulation(days)	2(1.25, 2)	1(1, 2)	0.034§
Time to removal of drainage(days)	5(4, 5)	4(3, 5)	0.002§
Postoperative hospitalization days	6(5, 7)	5(5, 6)	0.02§
**Follow-up data**			
Duration of follow-up	31.2(20, 41)	26.6(19, 34)	
BP improvement ratio (n%)	27(90.0)	16(88.9)	0.7144

Continuous normally distributed variables were expressed as the mean ± standard deviation (SD); Continuous non-normally distributed variables were expressed as the median (interquartile range); categorical variables are expressed as the number (percentage). Independent sample t-test was used to compare the differences between the means of two continuous normally distributed variables and Mann-Whitney U test was used to compare the differences between the means of two continuous non-normal distribution variables. Chi-square test was used to compare the differences between the categorical variables

*&* Independent sample t-test, *§* Mann-Whitney U test, *BP* Blood pressure, *CVD* Cardiovascular disease, *EBL* Estimated blood loss, *HI* Hemodynamic instability, *RLA* Retroperitoneal laparoscopic adrenalectomy, *RARLA* Robotic assisted retroperitoneal laparoscopic adrenalectomy

## Discussion

Once stimulated, a PHEO can secrete excess catecholamines, resulting in haemodynamic fluctuations that can cause perioperative mortality or severe morbidity. Therefore, adrenalectomy for the treatment of PHEO is an enormous challenge for surgeons, especially large PHEOs. RLA is a more optimized alternative to open adrenalectomy or transperitoneal laparoscopic adrenalectomy because of the small incision and the lack of mobilization of abdominal organs or intestines for the treatment of large PHEO. Robotic surgery, which represents the most advanced surgical technique in the world, has been reported in recent years for the treatment of PHEO [12–16]. However, most of these reported cases were small- to medium-sized PHEOs, and reports on the treatment of large PHEOs by robotic surgery have been scant. Therefore, we conducted this comparative study to evaluate the safety and feasibility of robotic surgery for the treatment of large PHEOs.

HI is a major concern in the resection of PHEO, especially large PHEO [17]. According to reports, the percentage of HI in the resection of PHEO ranges between 17 and 83% [18], which is consistent with our results. One of the most important findings in our study was that the incidence of HI in the RARLA group was significantly lower than that in the RLA group. Several studies have shown that tumour manipulation is one of the major stimulants causing a surge of catecholamines in the body, leading to HI [19–21]. In fact, the precision of robotic surgery and the elimination of vibrations reduces the likelihood that the tumour will be squeezed during the operation; therefore, intraoperative BP fluctuations will be minimal.

No significant difference in operative time between the two procedures was found in our study. Notably, robotic surgery has been performed in our centre only in recent years, while laparoscopic surgery has been used for a long time; thus, robotic surgery may not be as sophisticated as laparoscopic surgery to some extent. Therefore, robotic surgery still has the potential to require less operative time than laparoscopic surgery in the future since the robotic articular instruments, combined with a high-definition three-dimensional view and a more stable camera platform, allow for faster dissection [22–24]. Intraoperative blood loss is a good indicator of surgical quality. An exciting finding in our study is that the RARLA group had less blood loss and a lower transfusion rate than the RLA group, although no statistically significant difference in the intraoperative transfusion rate was identified between the two groups. The difference in intraoperative blood loss can be explained by the following reasons. The robot’s flexible arm and magnified HD stereo vision can reveal the deep anatomy and separation more precisely, and intraoperative bleeding is easier to find and control. Especially for PHEO, a large tumour with an abundant blood supply, fine operation is particularly important for bleeding control.

Our results showed that the rate of complications was similar between the RARLA and RLA groups, but postoperative recovery was faster in the RARLA group than in the RLA group. One of the interesting findings in our study was that the average postoperative anaesthetic use time in the robot group was shorter than that in the laparoscopic group. Although the difference was not statistically significant, postoperative anaesthetic use time in the robot group was 1 d shorter, indicating that robotic surgery causes less postoperative discomfort than laparoscopic surgery, which is consistent with the result of a comparative study conducted by Aliyev et al. [22]. The postoperative BP improvement rate reflects the effect of surgical resection of PHEO, and the rate in the RARLA group was similar to that in the RLA group during long-term postoperative follow-up.

Robotic surgery has some inherent drawbacks, such as a lack of haptic feedback, high purchase and maintenance costs, and large equipment that is difficult to move [23, 25–29]. However, these defects can be offset by more stable intraoperative haemodynamics, less intraoperative bleeding, and a faster postoperative recovery. Increasing the use of robots can reduce maintenance costs, which can be spread out across cases.

Transabdominal and retroperitoneal approaches are used for robot-assisted urological surgery. Retroperitoneal approaches are the most familiar surgical approaches for urologists, and most urologists prefer retroperitoneal approaches. However, some defects are associated with the retroperitoneal approach, the most important of which is the narrow operating space [14, 30]. Due to the large volume of the robotic arm, the requirements for the operating space and the layout of the cannula in robotic surgery are relatively strict. One issue to be aware of in our study is that the largest tumour diameter among all patients with PHEOs included in our study was 12 cm; thus, all the conclusions above are limited to patients with a PHEO diameter between 6 and 12 cm.

Similar to other comparative retrospective studies, several limitations exist in our study. First, this is a single-centre retrospective analysis prone to bias, and a multicentre prospective study is urgently needed to further validate our findings. Second, due to the rarity of large PHEOs and the limited number of robotic devices in our centre, the sample size of our study was low, which reduced the reliability of the final results to some extent. Third, intraoperative data, such as BP, heart rate, operation time and blood loss, were collected by consulting the anaesthesia record sheet. The data on the anaesthesia record sheet were updated every 5 min, and the intraoperative data were therefore not absolutely accurate, which may affect the final results.

## Conclusions

Compared with RLA for the treatment of large PHEOs, RARLA can result in more stable intraoperative haemodynamics, less intraoperative blood loss, and a faster postoperative recovery while achieving a similar BP improvement rate.

## Data Availability

The datasets used and/or analysed in the present study are available from the corresponding author upon reasonable request.

## Abbreviations

ASA: American Association of Anesthesiologists
BMI: Body mass index
BP: Blood pressure
CVD: Cardiovascular disease
CHD: Coronary heart disease
EBL: Estimated blood loss
HD: High definition
HI: Haemodynamic instability
NNIS: National Nosocomial Infections Surveillance
PHEO: Pheochromocytoma
RARLA: Robot-assisted retroperitoneal laparoscopic adrenalectomy
RLA: Retroperitoneal laparoscopic adrenalectomy
